# Dexmedetomidine in Patients Undergoing Transsphenoidal Resection of Pituitary Adenoma: An Updated Systematic Review and Meta-Analysis of Randomized Placebo-Controlled Trials

**DOI:** 10.7759/cureus.44132

**Published:** 2023-08-25

**Authors:** Nasser Aldosari, Shahad Alrashid, Anwar H Alshareeda, Abdulaziz Alenezi, Mohammad Y Alenezi, Abdulrahman Almutairi, Yousef Aldweesan, Fay Almajed, Abdulrazzaq Alshakhri, Fai Alwahhabi, Safwan A Almehmadi, Wardah Albzea, Mahmoud A Alsakka, Raghad Alhajaji

**Affiliations:** 1 Medicine and Surgery, Kuwait Institute for Medical Specializations, Kuwait City, KWT; 2 Pediatrics, Alsabah Hospital, Ministry of Health, Kuwait City, KWT; 3 Medical School, Umm Al-Qura University, Makkah, SAU; 4 Internal Medicine, Faculty of Medicine, Alexandria University, Alexandria, EGY; 5 Otorhinolaryngology and Facial Plastic Surgery, Canadian Medical Center, Kuwait City, KWT; 6 Family Medicine, Alhajj Primary Health Care, Ministry of Health, Makkah, SAU

**Keywords:** neuro-surgery, hr: heart rate, transsphenoidal resection, pituitary adenoma, dexmedetomidine

## Abstract

Dexmedetomidine has been widely studied in many surgical settings, with possible benefits in lowering anesthetic requirements, improving perioperative hemodynamic stability, and improving postoperative outcomes. This systematic review aims to evaluate the effects of dexmedetomidine in patients undergoing transsphenoidal resection of pituitary adenoma, shedding light on its potential as an adjunctive agent in anesthesia for this specific surgical population. In this review, we searched PubMed, Cochrane Library, Scopus, Web of Science, and Google Scholar from inception to July 20, 2023. A total of six randomized clinical trials (RCTs) investigating the effects of dexmedetomidine versus placebo in patients undergoing transsphenoidal resection of pituitary adenoma were included in this review. The outcomes of interest were extracted from the included studies as mean difference (MD) and standard deviation (SD), then analyzed using the Review Manager (RevMan, RevMan International Inc., New York, USA) software. Our literature search process retrieved 274 records. Of them, six studies were included in the meta-analysis. There was a significant difference between the dexmedetomidine group compared to the placebo group in terms of heart rate at the end of the surgery (MD = -16.5; CI = [-25.36 to -7.64]; P value = 0.0003) and after extubation (MD = -16.81; CI = [-23.18 to -10.43]; P values < 0.00001). Furthermore, dexmedetomidine significantly reduced the mean arterial blood pressure (MAP) at after both intubation and extubation (MD = -9.11 and -21.5; CI = [-13.56 to -4.65] and [-30.93 to -12.06]; P values < 0.00001). This systematic review and meta-analysis demonstrated that dexmedetomidine appears to have several potential benefits in patients undergoing transsphenoidal resection of pituitary adenoma. The use of dexmedetomidine was associated with reductions in heart rate, mean arterial blood pressure, blood loss, and duration of surgery, while showing no significant difference in propofol dose or time to extubation of the trachea.

## Introduction and background

Pituitary adenoma, a common benign tumor of the pituitary gland, and a major healthcare burden worldwide, constitutes a significant portion of intracranial neoplasms and accounts for approximately 10-15% of all primary brain tumors [1]. The predicted prevalence of pituitary adenomas is based on autopsy and radiographic data. The prevalence varies depending on the study and the source of information [2]. According to a meta-analysis, pituitary adenomas were found in 16.7% of autopsies, 14.4% of autopsies, and 22.5% of radiology tests [3]. Several population-based studies from various geographic areas have been conducted over many years to analyze the epidemiology of pituitary adenomas. The most widely accepted frequency is 115 per 100,000 people [2,4-6].

The management of pituitary adenoma is complex and multifaceted, involving various treatment modalities such as surgical resection, medical therapy, and radiation therapy, depending on tumor size, location, and hormonal activity [2,6]. Among these approaches, transsphenoidal surgical resection remains the backbone of treatment for these tumors [2,6-8]. However, transsphenoidal resection presents unique challenges that necessitate meticulous anesthetic control to maintain optimal surgical circumstances and patient safety [7,9]. Anesthetists have long attempted to improve the perioperative care of patients having transsphenoidal resection by investigating adjuvant medications with sedative and analgesic characteristics while maintaining hemodynamic stability [9-11]. The use of adjuvant drugs during anesthesia is not just for the transsphenoidal but has also been investigated in many head and neck surgeries [12-14].

Dexmedetomidine, a highly selective alpha-2 adrenergic agonist, has been identified as a promising adjuvant in anesthetic practice due to its distinct pharmacological profile [15]. It acts on presynaptic alpha-2 receptors in the central nervous system, resulting in decreased norepinephrine release and, as a result, sedation, anxiolysis, and analgesia [16]. Dexmedetomidine has been widely studied in many surgical settings, with possible benefits in lowering anesthetic requirements, improving perioperative hemodynamic stability, and improving postoperative outcomes [16,17].

Despite the potential benefits of dexmedetomidine, its role as an adjuvant in pituitary adenoma surgery remains a subject of ongoing investigation. While some studies have found that dexmedetomidine had a favorable effect [18-21], others have found inconsistent results or discovered limitations in specific patient populations [20]. Therefore, a comprehensive review of the current evidence is required to synthesize the existing data and offer an evidence-based assessment of the efficacy and safety of dexmedetomidine in this situation. This systematic review aims to fill this knowledge gap by critically evaluating the effects of dexmedetomidine in patients undergoing pituitary adenoma transsphenoidal resection, shedding light on its potential as an adjunctive agent in anesthesia for this specific surgical population.

## Review

Methods

This systematic review and meta-analysis follow the Preferred Reporting Items for Systematic Reviews and Meta-Analyses (PRISMA) guidelines [22] to ensure transparent and comprehensive reporting of the review process. Also, this study follows the Cochrane Handbook for Systematic Reviews and Meta-analysis of Intervention [23]. 

Eligibility Criteria

Our participants, intervention, comparison, outcome, and study design (PICOS) criteria comprised: (P): patients undergoing transsphenoidal resection of pituitary adenoma, (I): dexmedetomidine, (C): placebo, (O): efficacy endpoints, (S): randomized placebo-controlled trials (RCTs). There were no restrictions based on age, gender, or ethnicity. Studies with other interventions or no control group were excluded. 

Information Sources and Search Strategy

A comprehensive search of the electronic databases PubMed, Cochrane Library, Scopus, Web of Science, and Google Scholar was conducted to identify relevant studies published from inception to July 20, 2023. The search strategy was ((MPV-1440 OR MPV 1440 OR MPV1440 OR Precedex OR Dexmedetomidine OR Dexmedetomidine OR Dexmedetomidinum OR Medetomidine) AND (Pituitary Neoplasm OR Pituitary Tumor OR Pituitary Adenoma) AND (Transsphenoidal OR Trans-sphenoidal OR Trans sphenoidal OR Transsphenoid OR Trans sphenoid OR Trans-sphenoid)). The search was supplemented by hand-searching reference lists of relevant articles and grey literature to minimize publication bias.

Selection Process

Two independent reviewers screened the titles and abstracts of identified studies to assess their eligibility based on the predefined inclusion and exclusion criteria. Discrepancies were resolved through discussion. Full-text articles of potentially eligible studies were then obtained and assessed for final inclusion.

Data Collection Process and Data Items

Data from the included studies were independently extracted by two reviewers using a standardized data extraction form. The extracted information included study characteristics (authors, publication year, country, study design, and sample size), participant characteristics (age, gender, the American Society of Anesthesiologists, and the body mass index), intervention and comparison details (e.g., dosage and administration of dexmedetomidine and placebo), and outcome data (e.g., mean and standard deviation for continuous outcomes, counts for dichotomous outcomes).

Quality Assessment

The risk of bias in the included RCTs was independently assessed by two reviewers using the Cochrane Collaboration's tool for assessing the risk of bias in randomized trials (ROB-2) [24]. The domains assessed included the randomization process, deviations from intended interventions, missing outcome data, measurement of outcome, selection of the reported results, and other biases. 

Data Synthesis and Analysis

Statistical analysis was performed using Review Manager (RevMan, RevMan International Inc., New York, USA) software. For continuous outcomes, the mean difference (MD) or standardized mean difference (SMD) with corresponding 95% confidence intervals (CIs) were calculated. The heterogeneity among studies was assessed using the Chi-square test and measured using the I-square test. The results were considered heterogeneous if the alpha level of the Chi-square test was less than 0.1 and I-square was more than 50%. Sensitivity analysis was performed to assess the robustness of the findings by excluding studies with a high risk of bias or other potential sources of heterogeneity. Subgroup analysis was conducted to compare the two groups at different time points, which are baseline, after the intubation, start of the surgery, end of the surgery, and after the extubation. We did not assess the publication bias because the number of included studies is less than ten RCTs.

Results

Literature Search Results

**Figure 1:**
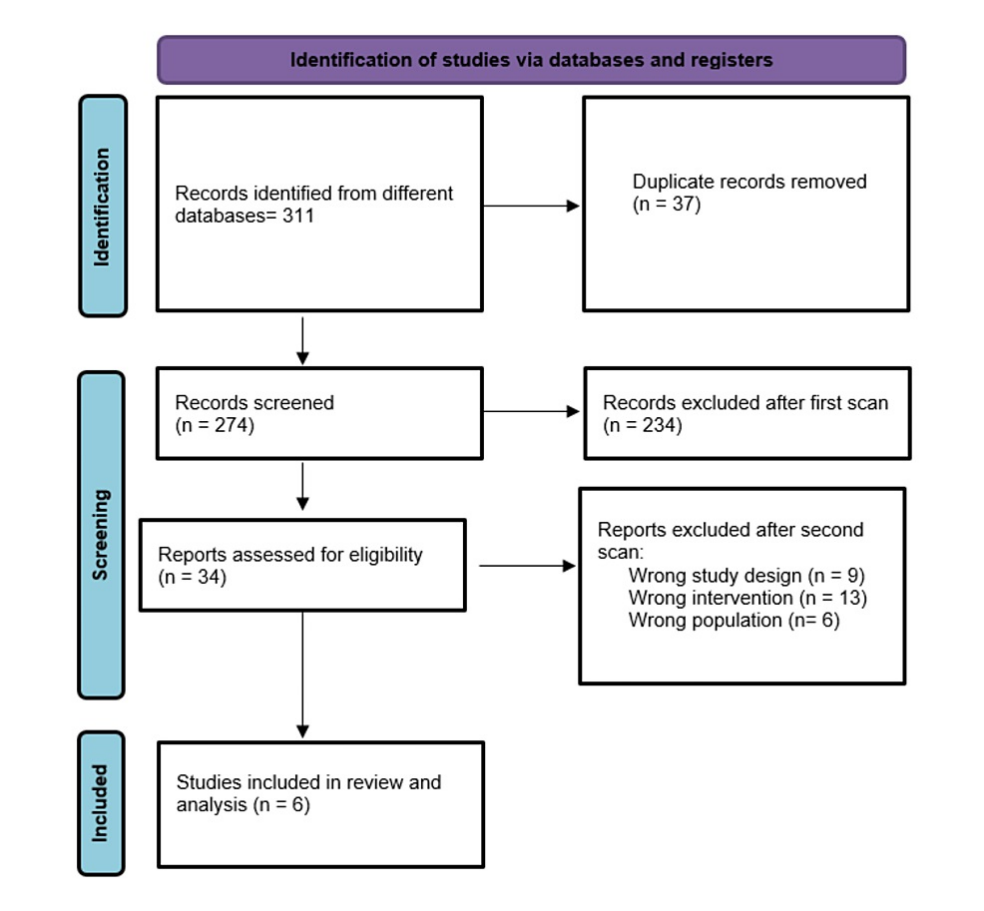
PRISMA flow diagram of studies’ screening and selection.

Our literature search process retrieved 274 records. Following title and abstract screening, 40 articles were eligible for full-text screening. Of them, 6 studies were included in the meta-analysis. The references of the included studies were manually searched, and no further articles were included. The PRISMA flow diagram of the study selection process is shown in Figure 1.

Characteristics of the Included Studies

The number of patients who were included in the meta-analysis was 290, who were randomly equally distributed into dexmedetomidine and placebo groups. A total of 125 (43.1%) of participants were males. A summary of the eligible studies and the characteristics of their patients are presented in Table 1 and Table 2, respectively. 

**Table 1:** Summary of the included studies. GH: growth hormone; ACTH: adrenocorticotropic hormone [18-21,25,26]

Study ID	Study design	Country	Number of participants	Loading dose of dexmedetomidine	Maintenance dose of dexmedetomidine	Pituitary gland status	Primary outcomes
Arefiev et al., 2020 [25]	prospective randomized trial	Russia	40	1 µg/kg/h over 10 minutes	0.7 mcg/kg/h	Prolactin and cortisol-releasing tumors	To study the efficacy and safety of using dexmedetomidine to stabilize hemodynamics and prevent arterial hypertension and agitation.
Bala et al., 2019 [26]	randomized, double-blinded	India	60	1 µg/kg was infused over 10 minutes	Not reported	Not reported	To evaluate the effects of dexmedetomidine administration on hemodynamic disturbances.
Gopalakrishna et al., 2015 [19]	randomized, double-blinded	India	44	1 mg/kg for 10 min	0.7 mg/kg/h	Nonfunctioning, GH-secreting, ACTH-secreting, and prolactin-secreting pituitary adenomas	The effect of dexmedetomidine on perioperative hemodynamics, anesthetic requirements, and recovery characteristics.
Mathew et al., 2020 [21]	randomized, double-blinded	India	40	1 µg/kg	0.5 µg/kg/h	Not reported	The effect of dexmedetomidine on hemodynamics, bleeding in the surgical field, and recovery parameters.
Kang et al., 2020 [18]	randomized, double-blinded	Korea	46	1 µg/kg over 10 minutes	0.2–0.7 mcg/kg/h	Non-functional	The effect of dexmedetomidine administration on intraoperative stress hormone release.
Salimi et al., 2014 [20]	randomized double-blinded	Iran	60	Not reported	0.6 µg/kg/hour	Not reported	The effect of dexmedetomidine on bleeding as well as surgeon’s satisfaction and hemodynamic stability.

**Table 2:** Baseline characteristics for the population of the included studies. ASA: American Society of Anesthesiologists; SD: Standard Deviation; N: Number; NR: Not Reported * Reported as median and inter-quartile range; # reported ASA status II/III [18-21,25,26]

Study ID	Number of patients		Age, Year (SD)		Gender (Male), N (%)		ASA Status I/II, N	
	Dexmedetomidine	Placebo	Dexmedetomidine	Placebo	Dexmedetomidine	Placebo	Dexmedetomidine	Placebo
Arefiev et al., 2020 [25]	20	20	53.5 (12.2)	53.1 (9.3)	9 (45)	10 (50)	14/6^#^	18/2^#^
Bala et al., 2019 [26]	30	30	37.2 (11.0)	41 (13.4)	16 (53.3)	17 (56.7)	20/10	18/12
Gopalakrishna et al., 2015 [19]	22	22	41.9 (10.4)	48.1 (12.3)	10 (45.45)	15 (68.2)	12/10	9/13
Mathew et al., 2014 [21]	20	20	NR	NR	NR	NR	NR	NR
Kang et al., 2020 [18]	23	23	48 (43–62)*	55 (39–56)*	8 (34.8)	11 (50)	17/6	18/5
Salimi et al., 2014 [20]	30	30	42.76 (13.6)	43.85 (11.46)	14 (46.7)	15 (50)	NR	NR

Heart Rate

**Figure 2:**
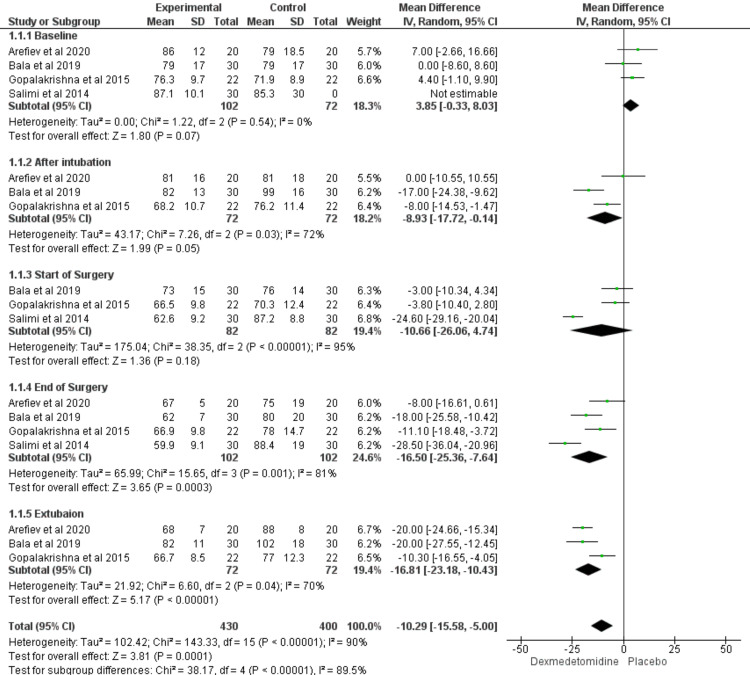
Forest plot showed the comparison between dexmedetomidine and placebo in patients undergoing transsphenoidal resection of pituitary adenoma in terms of heart rate. [18-21,25,26]

Four studies measured the heart rate at different points during the study. Our analysis showed that there was a significant difference at after intubation (MD = -8.93; CI = [-17.72 to -0.14]; P value = 0.05), end of the surgery (MD = -16.5; CI = [-25.36 to -7.64]; P value = 0.0003), and after extubation (MD = -16.81; CI = [-23.18 to -10.43]; p values < 0.00001). The analysis showed heterogeneity in the results at all-time points (I2 > 50%; P value > 0.1) except at baseline (I2 = 0%; P value = 0.54) (Figure 2).

Mean Arterial Blood Pressure

**Figure 3:**
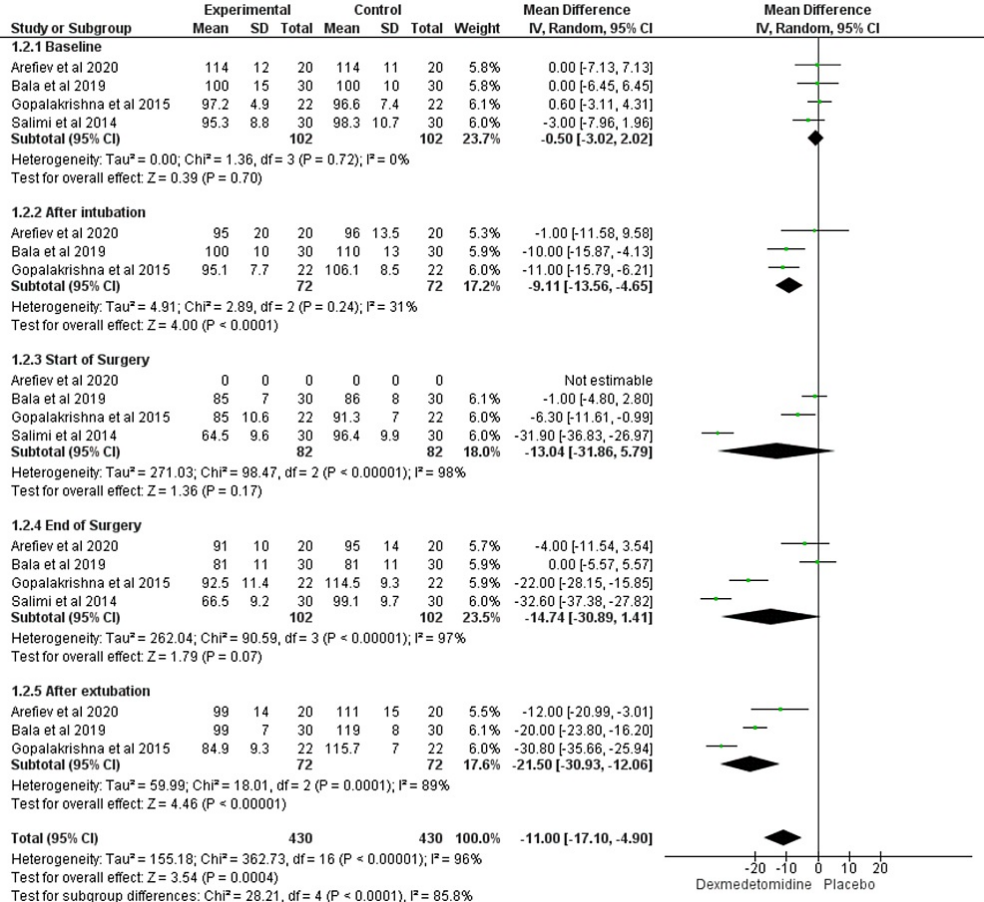
Forest plot showed the comparison between dexmedetomidine and placebo in patients undergoing transsphenoidal resection of pituitary adenoma in terms of MAP. MAP: mean arterial blood pressure [18-21,25,26]

Compared with the placebo group, dexmedetomidine significantly reduced the mean arterial blood pressure (MAP) at after both intubation and extubation (MD = -9.11 and -21.5; CI = [-13.56 to -4.65] and [-30.93 to -12.06]; P values < 0.00001). However, there was no significant difference between the two groups at the start of the surgery, and at the end of the surgery (P value > 0.05). The included studies were homogenous at baseline and after intubation (I2 = 0% and 31%; P values = 0.72 and 0.42; respectively), while they were heterogeneous at the rest time points (I2 > 50%; P value > 0.1) (Figure 3).

Duration of Surgery (min)

**Figure 4:**
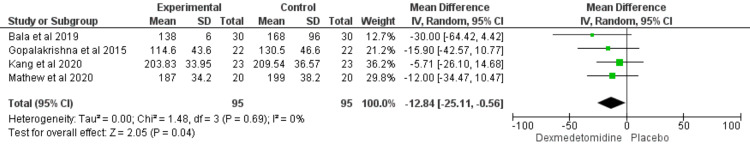
Forest plot showed the comparison between dexmedetomidine and placebo in patients undergoing transsphenoidal resection of pituitary adenoma in terms of duration of surgery (min). [18,19,21,26]

Four studies reported the duration of surgery. The analysis revealed that the dexmedetomidine group needed less time than the control group (MD = -12.84; CI = [-25.11 to -0.56]; P value= 0.04). The results were homogenous (I2 = 0%, P value = 0.69) (Figure 4).

Propofol Dose (mg/kg/h)

**Figure 5:**
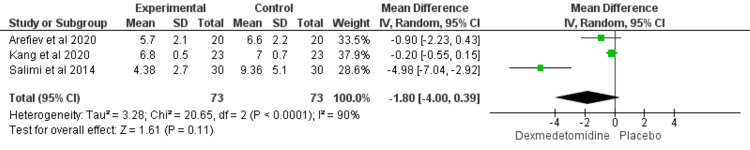
Forest plot showed the comparison between dexmedetomidine and placebo in patients undergoing transsphenoidal resection of pituitary adenoma in terms of propofol dose (mg/kg/h). [18,20,25]

**Figure 6:**
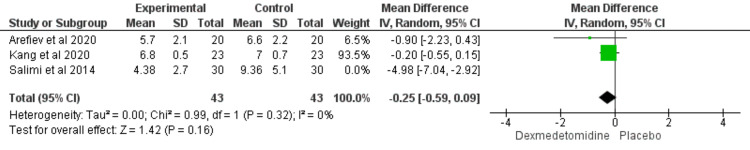
Forest plot with sensitivity analysis showed the comparison between dexmedetomidine and placebo in patients undergoing transsphenoidal resection of pituitary adenoma in terms of propofol dose (mg/kg/h). [18,20,24]

The propofol dose did not significantly differ between the two groups (MD = -1.8; CI = [-4 to 0.39]; P value= 0.11), and the results were heterogeneous (I2 = 90%; P values < 0.00001) (Figure 5). However, we resolved the heterogeneity by excluding the Salimi et al. study [20] from the combined results (I2 = 0%; P value = 0.32) but the result is still insignificant (MD = -0.25; CI = [-0.59 to 0.09]; P value = 0.16) (Figure 6).

Blood Loss (ml)

**Figure 7:**
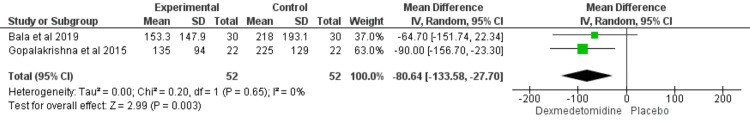
Forest plot showed the comparison between dexmedetomidine and placebo in patients undergoing transsphenoidal resection of pituitary adenoma in terms of blood loss (ml). [19,26]

Our analysis reported that the control group significantly lost blood more than the dexmedetomidine group (MD = -80.64; CI = [-133.85 to -27.7]; P value = 0.003) and the results were homogenous (I2 = 0%; P value = - 0.65) (Figure 7).

Time to Extubation of Trachea (min)

**Figure 8:**
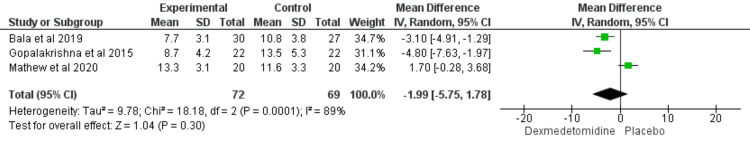
Forest plot showed the comparison between dexmedetomidine and placebo in patients undergoing transsphenoidal resection of pituitary adenoma in terms of time to extubation of trachea (min). [19,21,26]

Three studies reported the time required to extubation the trachea. Our results showed that there was no significant difference between the two groups (MD = -1.99; CI = [-5.75 to 1.78]; P value = 0.3). The studies included in the analysis were heterogeneous (I2 = 89%; P value = 0.0001) (Figure 8). However, after the sensitivity analysis and removing the Mathew et al. study [21], the results became homogenous (I2 = 0%; P value = 0.32) with significant difference between the groups (MD = -3.6; CI = [-5.12 to -2.07]; P value < 0.00001).

Modified Aldrete Score

**Figure 9:**
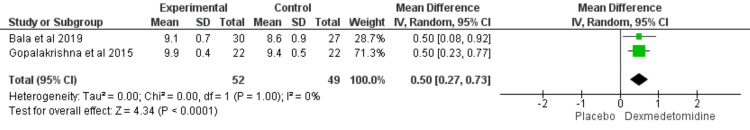
Forest plot with showed the comparison between dexmedetomidine and placebo in patients undergoing transsphenoidal resection of pituitary adenoma in terms of Modified Aldrete score. [19,26]

The dexmedetomidine group had significantly higher score compared to the placebo group (MD = 0.5; CI = [0.27 to 0.73]; P value < 0.00001) and the results were homogenous (I2 = 0%; P value = 1) (Figure 9).

Assessment of the Bias

**Figure 10:**
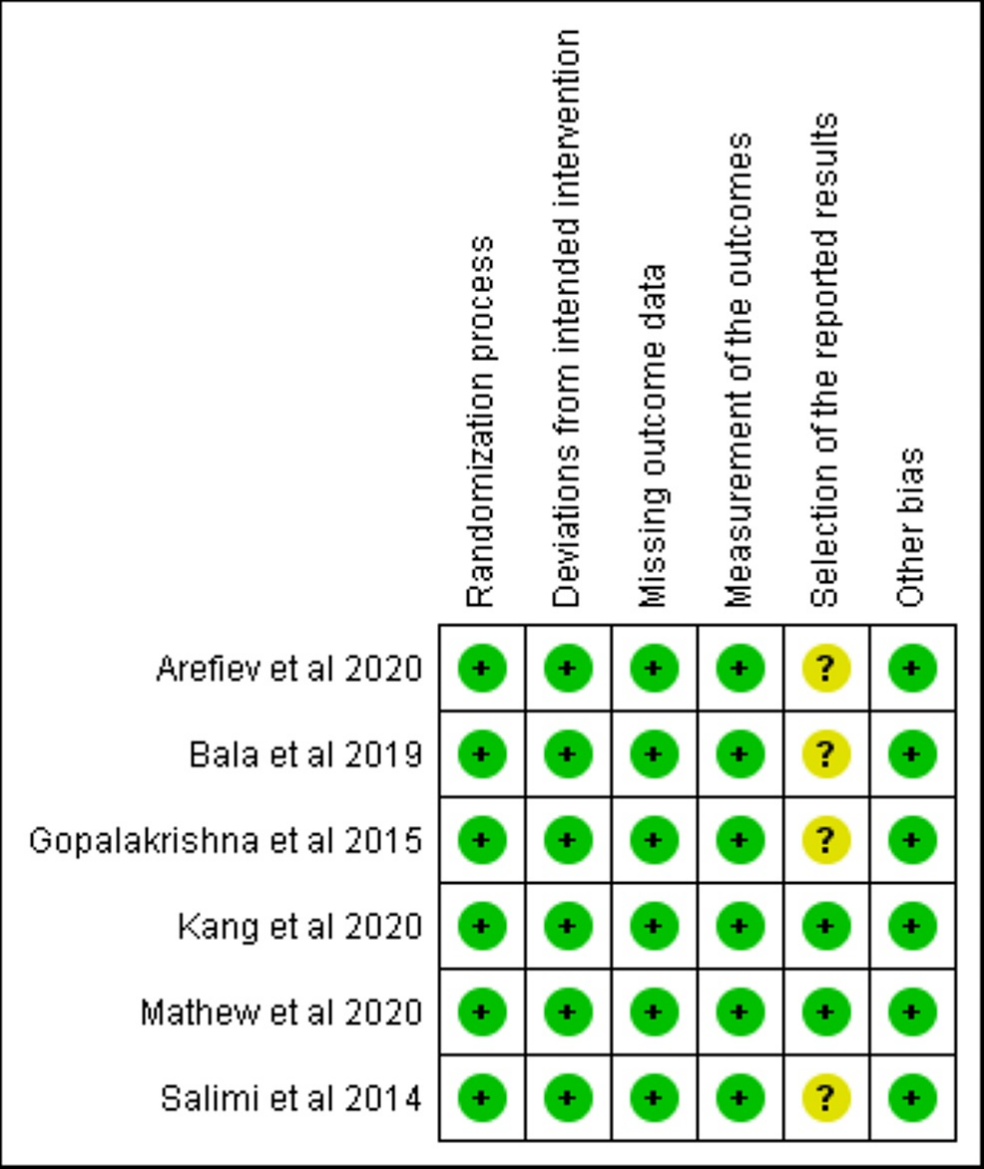
Risk of bias summary. [18-21,25,26]

Out of the studies included in our meta-analysis, four studies reported uncertainty in terms of the selection of reported results. Therefore, all included studies showed an overall low risk of bias (Figure 10).

Discussion

Significance of the Study

To the best of our knowledge, this is the most up-to-date systematic review and meta-analysis that evaluates the efficacy and safety of dexmedetomidine in patients who have undergone transsphenoidal surgical resection of pituitary adenoma. This meta-analysis put a stone in the build of the dexmedetomidine benefits as an adjuvant anesthetic agent during surgeries in general, not just the transsphenoidal surgical resection of pituitary adenoma.

Summary of Findings

The meta-analysis included six randomized clinical trials with a total of 290 patients. Dexmedetomidine showed a significant difference in heart rate reduction compared to placebo after intubation, at the end of surgery and extubation, but not at other time points, and significantly reduced MAP after both intubation and extubation, but not at other time points. Moreover, dexmedetomidine was associated with a significantly shorter duration of surgery and lower intraoperative blood loss compared to placebo. However, there was no significant difference in propofol dose, or the time required for tracheal extubation between the two groups, but sensitivity analysis indicated a significant difference after removing one study. Finally, the dexmedetomidine group had a significantly higher Modified Aldrete score compared to the control group.

Explanation of the Findings

This systematic review and meta-analysis provide important insights into the effects of dexmedetomidine in patients undergoing transsphenoidal resection of pituitary adenoma. The observed decrease in heart rate after administration of dexmedetomidine is consistent with its well-established mechanism of action as an alpha-2 adrenergic agonist [27]. Dexmedetomidine reduces heart rate via modifying sympathetic tone [28,29], which reduces the consequences of stress responses during surgery. This effect is most pronounced at the end of surgery and during extubation, when the reduction in sympathetic outflow can contribute to a more gradual emergence from anesthesia, thereby reducing the risk of hemodynamic fluctuations [18-21,25,26].

The alpha-2 agonist characteristics of dexmedetomidine can also be responsible for the dexmedetomidine group's considerable decrease in mean arterial blood pressure during intubation and extubation [27]. Dexmedetomidine lowers systemic vascular resistance by attenuating norepinephrine release [30], which lowers blood pressure. This impact is especially important during the perioperative phase when blood pressure changes can have serious consequences for brain perfusion and general hemodynamic stability [31-33]. The temporary nature of the dexmedetomidine's hypotensive impact and the drug's effects wearing off with time [16,17] may be the cause of the lack of a meaningful difference in MAP at subsequent time points. The sedative effects of dexmedetomidine [15,17] may have contributed to the reduced length of operation. Inducing a state of conscious sedation with dexmedetomidine may result in less patient movement and better compliance during surgery [34]. As a result of better surgical circumstances and a more expeditious technique, this can result in shorter operating times. Dexmedetomidine's sedative impact is consistent with its known characteristics and with results from other surgical procedures [35-38] where it has been demonstrated to improve patient comfort and surgeon satisfaction [39,40].

Dexmedetomidine has been shown to significantly reduce intraoperative blood loss, which may be due to its vasoconstrictive characteristics [41,42]. This effect is very important in procedures like transsphenoidal resection, where limiting blood loss helps improve sight and surgical accuracy [43,44]. Dexmedetomidine's potential to improve intraoperative circumstances by lowering blood loss is consistent with its status as an adjuvant with advantageous hemodynamic effects. The lack of a significant difference in propofol dose between the dexmedetomidine and placebo groups implies that while dexmedetomidine may have benefits for hemodynamic stability and sedation, it has little to no effect on the total amount of anesthetic needed. Given the observed heterogeneity in the propofol dose results, more research is required to determine the best dexmedetomidine dosing regimens in this situation, taking into account patient characteristics and surgical nuances.

Finally, a higher Modified Aldrete score in the dexmedetomidine group indicates better postoperative recovery. This data implies that the sedative and analgesic characteristics of dexmedetomidine lead to improved patient comfort and readiness for release from the post-anesthesia care unit. A higher Modified Aldrete score demonstrates dexmedetomidine's capacity to allow easier transitions from anesthesia to alertness [45], encouraging a positive postoperative patient experience. Our study supports the use of dexmedetomidine as an adjuvant anesthesia medication for patients undergoing transsphenoidal resection of pituitary adenoma, which comes in agreement with the previous meta-analyses that recommended the usage of many adjuvant drugs, not only the dexmedetomidine, for anesthesia of different surgeries [46-48]. In addition, it comes in agreement with the previous meta-analyses that showed the beneficial effects of dexmedetomidine in different types of surgeries [35-40,49-51]. Moreover, a previous meta-analysis conducted by Liu et al. [52] evaluating the efficacy and safety of dexmedetomidine in patients who had undergone transsphenoidal surgical resection of pituitary adenoma, including four randomized controlled trials with a total of 160 patients showed that the dexmedetomidine significantly reduced mean arterial pressure and heart rate at 30 minutes, blood loss and fentanyl usage. Our meta-analysis supported their results except for the fentanyl usage, as our results showed that no significant difference in propofol dose was observed between the dexmedetomidine and placebo groups. However, our results are the most updated with a larger number of included studies with a larger sample size (290 patients).

Strength Points and Limitations

Our study is the most up-to-date systematic review and meta-analysis that evaluates the efficacy and safety of dexmedetomidine in patients who have undergone transsphenoidal surgical resection of pituitary adenoma. Our study overcame the previous meta-analysis by including a larger number of studies with a larger sample size. In addition, we performed a subgroup analysis according to the time, which allowed us to investigate the dexmedetomidine hemodynamic effects by time. However, some limitations should be considered. Firstly, the number of included studies may limit the ability to detect publication bias. Secondly, heterogeneity was observed in several outcomes, which may have been influenced by variations in study designs and patient characteristics. Nevertheless, sensitivity and subgroup analyses were performed to address these issues. Lastly, the overall quality of evidence may be influenced by the quality of the included studies, and more high-quality RCTs are needed to strengthen the conclusions.

Recommendations for Future Research and Clinical Practice

On the basis of the results of this meta-analysis, we recommend clinicians consider incorporating dexmedetomidine into their anesthesia management for patients undergoing transsphenoidal resection of pituitary adenoma. However, additional well-designed randomized controlled trials with larger sample sizes are required to validate the results and investigate the optimal dosage and administration of dexmedetomidine in this context. For a greater understanding of dexmedetomidine effects, future research could look into long-term outcomes including postoperative pain, nausea, and patient satisfaction

## Conclusions

This systematic review and meta-analysis demonstrated that dexmedetomidine appears to have several potential benefits in patients undergoing transsphenoidal resection of pituitary adenoma. The use of dexmedetomidine was associated with reductions in heart rate, mean arterial blood pressure, blood loss, and duration of surgery, while showing no significant difference in propofol dose or time to extubation of trachea. These data imply that dexmedetomidine could be a useful adjuvant in optimizing anesthetic management in this patient population. However, more research is required to confirm these findings and develop effective dosing regimens.
